# Hepatitis E Virus among Persons Who Inject Drugs, San Diego, California, USA, 2009–2010

**DOI:** 10.3201/eid1910.130630

**Published:** 2013-10

**Authors:** Reena Mahajan, Melissa G. Collier, Saleem Kamili, Jan Drobeniuc, Jazmine Cuevas-Mota, Richard S. Garfein, Eyasu Teshale

**Affiliations:** Centers for Disease Control and Prevention, Atlanta, Georgia, USA (R. Mahajan, M.G. Collier, S. Kamili, J. Drobeniuc, E. Teshale);; University of California School of Medicine, San Diego, California, USA (J. Cuevas-Mota, R.S. Garfein)

**Keywords:** hepatitis E virus, viruses, hepatitis E, injection drug use, persons who inject drugs, seroprevalence, antibodies, IgG, San Diego, California

## Abstract

Data about prevalence of hepatitis E virus infection in persons who inject drugs are limited. Among 18–40-year-old persons who inject drugs in California, USA, prevalence of antibodies against hepatitis E virus was 2.7%. This prevalence was associated with age but not with homelessness, incarceration, or high-risk sexual behavior.

Serologic evidence of hepatitis E virus (HEV) infection (i.e., IgG against HEV) in the United States has been reported to be ≤21% on the basis of national estimates during 1988–1994 (*1*). Among marginalized populations, such as persons who inject drugs (PWID) and homeless or incarcerated persons, HEV infection ranges from 5% to 23%, although data have been limited for these groups (*2**–**6*). We determined the seroprevalence of and factors associated with IgG against HEV among 18–40-year-old PWID in San Diego, California, USA.

## The Study

Methods for the Study to Assess Hepatitis C Risk have been summarized (*7*). In brief, during March 2009–June 2010, persons 18–40 years of age who were residents of San Diego County, California, and who had injected drugs in the previous 6 months were recruited to participate in this study. Eligibility screening and acquisition of informed consent for potential participants were followed by a behavioral risk assessment and serologic testing.

Data collected included participant demographics, substance use, injection practices, diagnosis with sexually transmitted infections, exchange of sex for money, homelessness, travel to Mexico, and HIV status. Serologic testing included detection of antibodies against hepatitis A virus (HAV), hepatitis B virus core antigen, and hepatitis C virus (HCV) by using the VITROS Immunodiagnostic System (Ortho Clinical Diagnostics, Rochester, NY, USA), and IgG against HEV by using a commercial assay (DSI, Saronno, Italy).

We performed a comparative analysis of all persons on the basis of their status for IgG against HEV by using demographics, seropositivity for other viral hepatitides, travel to Mexico, history of incarceration, homelessness, HIV status, and high-risk sexual behavior. We used bivariate logistic regression to calculate odds ratios; 95% CIs; and p values, which were set at 0.05 to determine significance for factors associated with HEV prevalence. All data were analyzed by using SAS version 9.2 (SAS Institute, Cary, NC, USA).

Of 508 PWID, 72% were men, their mean age was 29 years (range 18–40 years); and 62% were white. Fourteen (2.7%) persons had IgG against HEV; none of these persons were positive for HEV RNA by PCR (all were negative for IgM against HEV). Of the 14 persons with IgG against HEV, 11 (79%) were men; their mean age was 33.4 years (range 30–36 years); and 57% were white (Table). Relative to participants <30 years of age, persons ≥30 years of age were more likely to be positive for IgG against HEV (odds ratio 3.61, 95% CI 1.31–9.94). Travel history and presence of antibodies against HAV, hepatitis B virus, or HCV were not associated with presence of antibody against HEV. Bivariate logistic regression showed that there was no association between presence of IgG against HEV and a history of incarceration, sharing of injection drug equipment, homelessness, high-risk sexual behavior, and HIV status.

**Table:** Prevalence of IgG against hepatitis E virus among persons who inject drugs, San Diego, California, USA, 2010*

Characteristic	Positive for IgG against HEV, n = 14	Negative for antibody against HEV, n = 494†	p value
Sex			
M	11 (79)	357 (72)	0.707
F	3 (21)	137 (28)	ND
Mean age, y (95% CI)	33.4 (30.1–36.6)	28.5 (27.9–29.0)	<0.003
Age ≥30 y	9 (64)	170 (34)	0.013
Race			0.776
White	8 (57)	272 (55)	ND
Black	2 (14)	34 (7)	ND
Hispanic	2 (14)	137 (28)	ND
Other	1 (7)	15 (3)	ND
Homeless	11 (79)	287 (58)	0.326
History of incarceration	12 (86)	376 (76)	0.263
Sharing any drug injecting equipment	8 (57)	354 (72)	0.587
Diagnosis of sexually transmitted infection†	1 (7)	93 (19)	0.443
Exchange of sex for money	5 (36)	142 (29)	0.308
Travel to Mexico	10 (71)	314 (64)	0.373
Antibody against HAV	3 (21)	190 (38)	0.504
Antibody against HBc	3 (21)	201 (41)	0.138
Antibody against HCV	3 (21)	128 (26)	0.351
HIV positive	1 (7)	21 (4)	0.751

*Values are no. (%) unless otherwise indicated. ND, not determined; HAV, hepatitis A virus; HBc, hepatitis B core antigen; HCV, hepatitis C virus.  †Totals may not equal 100% because of missing data.

## Conclusions

We found an overall HEV seroprevalence of 2.7% in young PWID in the United States. This seroprevalence was higher among participants ≥30 years of age than in participants <30 years of age. Variables typically associated with HCV/HIV transmission (i.e., high-risk sexual behavior, incarceration, or sharing of injection drug use equipment) were not associated with presence of antibodies against HEV. These findings were consistent with results of a study that found no association between antibodies against HEV and co-infection with other hepatitis viruses or sharing of drug paraphernalia (*2*).

Because of the common mode of fecal–oral transmission of HAV and HEV, other studies have also investigated an association between HAV and HEV infections, but results have been inconclusive (*1**,**5**,**6*). As in previous studies, we found an association of presence of antibodies against HEV and age (*1**,**6*). Higher prevalence among older PWID suggests that there may be age-related exposures independent of injection drug use that increases the likelihood of HEV infection. This birth cohort effect has been seen in other low-prevalence countries, such as Denmark (*8*), and decreased possible exposure may help explain the lower prevalence rates in our study.

This study had a few limitations. Our small sample size reduced the potential to detect significant differences between HEV-negative and HEV-positive persons. In addition, we did not have information about other exposures that have been associated with HEV infection, including particular dietary or zoonotic exposures or history of travel to a country to which HEV is endemic. Therefore, the potential effect of these exposures cannot be assessed. Information about HEV genotype was not available for seropositive persons, which might have provided clues as to the mechanism of exposure. Lower prevalence estimates may also reflect the fact that our population only included persons 18–40 years of age. Previous data have suggested that increasing age is associated with higher HEV positivity (*6*), particularly in countries in which prevalence is low and infection is caused mainly by HEV genotype 3 (*9*). Although our data cannot be generalized to the US population, seroprevalence in this study appears to be low, which is similar to time trends in the general population of other low-prevalence areas (*8*).

Variability in assay types used may account for discrepancies seen with previous seroprevalence studies of HEV. In a study evaluating the performance and concordance between various assays for detection of IgG against HEV available at the time, overall concordance ranged from 49% to 94% (median 69%), and concordance among reactive serum samples ranged from 0% to 89% (median 32%) (*10*).

Evaluation of the performance characteristics and concordance of currently available assays for detection of antibodies against HEV, including the assay used in this study, remains to be determined. Overall, our data showed an increase in antibodies against HEV for PWID ≥30 years of age and no other association with other reported risk factors. Future research is needed to explore other marginalized populations in HEV-endemic areas to determine whether there are other risk factors that have not been identified in low-prevalence areas.
